# Linkage analysis revealed risk loci on 6p21 and 18p11.2-q11.2 in familial colon and rectal cancer, respectively

**DOI:** 10.1038/s41431-019-0388-3

**Published:** 2019-04-05

**Authors:** Susanna von Holst, Xiang Jiao, Wen Liu, Vinaykumar Kontham, Jessada Thutkawkorapin, Jenny Ringdahl, Patrick Bryant, Annika Lindblom

**Affiliations:** Department of Molecular Medicine and Surgery, Karolinska Institutet, and Department of Clinical Genetics, Karolinska University Hospital, 171 76 Stockholm, Sweden

**Keywords:** Risk factors, Cancer genetics

## Abstract

Colorectal cancer (CRC) is one of the major cancer types in the western world including Sweden. However, known genetic risk factors could only explain a limited part of heritability of the disease. Moreover, colon and rectal cancers are habitually discussed as one entity, colorectal cancer, although different carcinogenesis has been recognized. A genome-wide linkage scan in 32 colon- and 56 rectal cancer families from Sweden was performed based on 475 non-FAP/HNPCC patients genotyped using SNP arrays. A maximum HLOD of 2.50 at locus 6p21.1-p12.1 and a HLOD of 2.56 at 18p11.2 was obtained for colon and rectal cancer families, respectively. Exome sequencing over the regions of interest in 12 patients from six families identified 22 and 25 candidate risk variants for colon and rectal cancer, respectively. Haplotype association analysis in the two regions was carried out between additional 477 familial CRC cases and 4780 controls and suggested candidate haplotypes possibly associated with CRC risk. This study suggested two new linkage regions for colon cancer and rectal cancer with candidate predisposing variants. Further studies are required to elucidate the pathogenic mechanism of these regions and to pinpoint the causative genes.

## Introduction

More than 1.2 million new cases are diagnosed with colorectal cancer (CRC) in the world yearly, primarily in the western world [1]. In Sweden, CRC is the third most common cancer type among women and men, and affects 4000–6000 individuals each year [2]. Less than 5% of CRC cases are caused by known genes, such as those causing familial adenomatous polyposis (FAP) and hereditary non-polyposis colorectal cancer (HNPCC) [3]. Previous CRC genes mapped using linkage analysis include *APC* [4], *MSH2* [5], and *MLH1* [6]. Other linkage studies have suggested potential CRC loci at 2q, 3q, 4q, 8q, 9q, 10p, 12q, 14q, and 15q [7–14]. Moreover, hundreds of common variants have been reported by genome-wide association studies (GWAS) to be associated to CRC, but they only describe a limited portion of disease risk [15, 16]. Altogether, germline variants in known genes and moderate- and low risk variants were suggested to explain 10–15% of the genetic CRC contribution [17]. Although parts of the causative CRC genetic factors are known, further investigation to learn about the missing genetics is important, since up to 35% of all CRC cases could be explained by hereditable factors [18].

Colon cancer and rectal cancer are habitually discussed as colorectal cancer. The question whether it is one single or two different entities has been under debate. Some studies have presented possible differences and recognized that colon and rectal cancer have different carcinogenesis. Bufill et al. reported that the location of the tumor might be a marker for the clinical feature [19]. Tumors arise predominantly distal to the splenic flexure in adenomatous polyposis, while in HNPCC, most tumors arise proximal to the splenic flexure [19]. One study disclosed a higher frequency in genetic alteration and allelic losses on chromosome 5q, 17p and 18 among distal compared to proximal colorectal tumors [20]. Kapiteijn et al. reported that rectal cancers had a significantly higher immunohistochemical expression of TP53 and nuclear β-catenin compared to colon cancers and that *TP53* mutation rate was higher in rectal cancer cases [21]. However, no significant differences were seen for clinical and histopathological data [21]. Another study showed that *KRAS* variants in stool DNA were associated with tumors in the sigmoid colon and rectum but not with tumors from other parts of the colon [22]. Elevated expression of the oncogene *MYC* was seen more often in the left-side (rectum, sigmoid and descending colon) compared to the right-side (caecum and ascending colon) of colorectum [23].

Therefore, novel loci harboring predisposing genes could possibly be found by analyzing colon and rectal cancer families separately. Thus, we performed a new linkage scan on 32 colon cancer and 56 rectal cancer families corresponding to 169 and 306 individuals, respectively. These families were included in a genome-wide linkage analysis of 121 families conducted previously [9]. In order to find evidence supporting the candidate regions revealed by linkage analysis and to further pinpoint genes and variants that potentially affect functions, we performed targeted exome sequencing over the two regions in the families mostly contributing to the positive LOD scores and haplotype association analysis in additional CRC cases and controls.

## Materials and methods

### Study subjects

Cancer families were recruited through the Department of Clinical Genetics at the Karolinska University Hospital in Stockholm, Sweden between 1990 and 2005. FAP was excluded using medical records from affected individuals and HNPCC was excluded using our current clinical protocols [24]. Families were included in the study if there were at least two affected relatives informative for linkage analysis (i.e., at least a sib-pair). A family was included in the linkage analysis if the family could be classified to have a risk for colon or rectal cancer. Eighty-eight of the previously analyzed 121 families fulfilled the criteria above and were included in the linkage analysis (Table 1).

**Table 1:** Cancer families included in the linkage analysis

	No. of families	No. of individuals genotyped	No. of affected genotyped	Mean age	Youngest age at diagnosis
Colon	32	306	108	65.8	33
Rectum	56	169	67	64	31

A case–control study used 477 familial CRC cases from the Swedish Colorectal Cancer Low-risk Study and 4780 control individuals from the Swedish Twin Registry [25, 26]. The 477 CRC cases were from a cohort of more than 3300 consecutive patients operated on for CRC in 14 hospitals in and around Stockholm and Uppsala between 2004 and 2009. For the twin controls, phenotypic data on cancer had previously been obtained through linking the twins to the Swedish Cancer Registry using the unique person identification number available for all Swedish citizens. Only one twin from each twin pair where none was affected was considered eligible for serving as control in the association analysis.

The study was undertaken in agreement with the Swedish legislation of ethical permission (2003:460) and according to the decision in the Stockholm regional ethical committee (2008/125-31.2 and 02-489). All participants had given informed consent to participate in the study.

### Genotyping and quality control (QC)

Genomic DNA was extracted from peripheral blood using standard procedures. Genotyping was performed as previously described [9].

In order to generate haplotypes for CRC families, a total of 60 parent–child pairs from 10 colon cancer families, 17 rectal cancer families as well as 33 CRC families without a clear tumor location predominance from this study was genotyped with the Illumina Infinium HumanOmniExpress-12v1 BeadChip (730,525 markers) at the SNP&SEQ Technology Platform in Uppsala, Sweden. The overall reproducibility of the genotype data was 99.996% based on 1.53% of duplicated genotyping, with an average call rate per SNP of 99.43%.

The 477 additional familial CRC cases were genotyped using the Illumina Infinium OncoArray-500K BeadChip at the Center for Inherited Disease Research at Johns Hopkins University, MD, US [16]. The 4780 controls from the Swedish TwinGene registry were genotyped using the Illumina OmniExpress BeadChip in Uppsala, Sweden. All samples went through initial QC at their corresponding centers before being merged on the 235,573 SNPs that were shared between the two platforms. QC of the merged dataset excluded variants from analysis if call rate was ≤97%, minor allele frequency was <1% or if the variant deviated significantly from Hardy–Weinberg equilibrium (*p* ≤ 1E−7). Samples were removed in case of genotyping success rate was <97%, gender discrepancy between reported and X-chromosome heterozygosity-predicted, abnormal heterozygosity (>3 standard deviations from mean) or detection of cryptic relatedness.

### Linkage analysis

Pedcheck [27] was used to check for the initial Mendelian inheritance analysis among the families. The family-based genetic model was used for parametric linkage analysis for all chromosomes including chromosome X. Non-parametric analysis was performed as a supplement. LOD scores as well as heterogeneity LOD scores were computed using MERLIN (version 1.1.2) [28] and was given for all genotyped positions. Analyses were done assuming both dominant and recessive traits and the parameters were set as described by our previously published paper [9]. Individuals with CRC or a polyp with high degree dysplasia were coded as affected. All subjects in the 88 families from the two genotyping sessions were included in the analysis. Due to two genotyping sets, two map files were merged and 7256 markers were used in the analysis. As a consequence of limitations in MERLIN, four large families had to be split when running the analysis.

### Exome sequencing and variant calling

Twelve patients from six families, three colon (110, 301, 350) and three rectal (8, 918, 1213) cancer families, respectively, were selected for exome sequencing based on their major contribution to the LOD scores in the linkage regions. In four families two affected sibs were sequenced. In one family a single patient was sequenced and in the last family three sibs were subject to sequencing.

Sequencing libraries were prepared from genomic DNA using TruSeq DNA Sample Preparation Kit (Illumina, San Diego, CA, USA) or SureSelectXT Reagent HSQ 96 Auto kit (Agilent, Santa Clara, CA, USA) according to manufacturers’ instructions. Exome enrichment was performed using TruSeq Exome Enrichment Kit (Illumina) or SureSelect XT Human All Exon V5 library (Agilent). Multiplexed paired-end libraries were pooled in equal molar and sequenced on an Illumina HiSeq 2000 or HiSeq 2500 system (Illumina) according to manufacturer’s instructions.

Base calling was performed on the instrument with RTA (1.12.4.2 or 1.13.48) and the resulting BCL files were filtered, de-multiplexed, and converted to FASTQ format using CASAVA 1.7 or 1.8 (Illumina). Raw reads were mapped to the hg19 GRCh37 reference genome sequence using bwa (0.5.9), and variants were called using GATK (1.0.5974) following realignment and recalibration. Variant annotation was performed using ANNOVAR (released 2013-08-23).

### Haplotype association analysis

Association analysis were carried out between 477 familial CRC cases and 4780 controls over the two regions of interest revealed by linkage analysis in sliding windows containing 1–25 consecutive markers. In short, haplotype frequency was estimated for each window and *p*-values were calculated using Plink v.1.07 [29].

### Data deposition

Non-synonymous coding sequence variants with a MAF < 0.20 that segregated in at least one of the six selected families with corresponding disease information were deposited to the gene variant database of Leiden Open Variation Database (https://databases.lovd.nl/shared/genes). Individual IDs were #00208599, #00208600, #00208601, #00208603, #00208611, and #00208612 for one representative from each of the families 310, 110, 350, 8, 918, and 1213, respectively.

## Results

### Linkage analysis suggested candidate regions for colon and rectal cancer separately

**Fig. 1:**
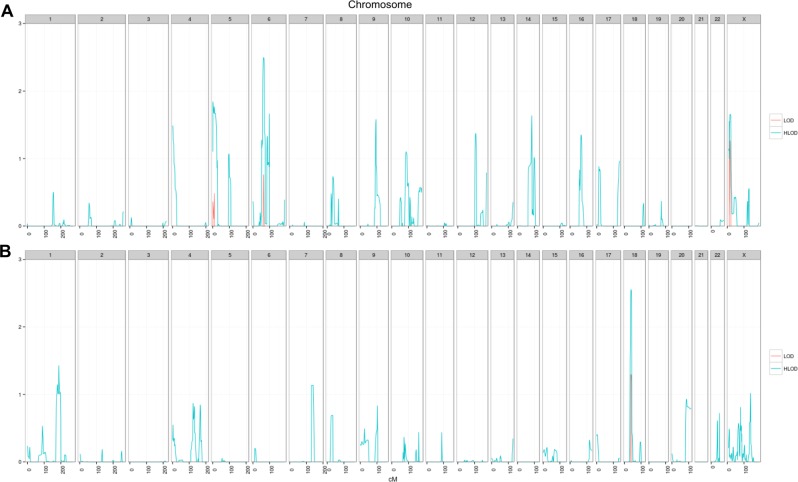
LOD/HLOD score plots for colon and rectal cancer families. **a** LOD/HLOD plot for 32 colon cancer families. **b** LOD/HLOD plot for 56 rectal cancer families. LODs are represented in red and HLODs are represented in cyan

A total of 88 families were genotyped and analyzed in two groups, comprising of 32 colon and 56 rectal cancer families with 306 and 169 individuals, respectively (Table 1). No LOD or HLOD score above three was observed. However, suggestive linkage could be demonstrated for colon as well as rectal cancer families (Fig. 1). Regions with HLODs above 1.0 are summarized in Table 2. A maximum HLOD of 2.5 was observed for a 6 Mb region at locus 6p21.1-p12.1 in the colon cancer families. The highest HLOD was 2.6 for the rectal cancer families at locus 18p11.2 with about 10 Mb in length.

**Table 2:** Linked regions with maximum observed HLODs above 1.0

Study group	Linked region	cM. SNP	Model	HLOD (*α*)
Colon	5p15.31	rs875347	Dominant	1.2 (0.8)
Colon	6p21.1	rs1537638	Dominant	1.4 (0.9)
Colon	19q13.13	rs241942	Dominant	1.0 (0.8)
Colon	4p16.3	rs935971	Recessive	1.5 (0.5)
Colon	5p15.3	rs413666	Recessive	1.8 (0.6)
Colon	6p21.1	rs722269	Recessive	2.5 (0.5)
Colon	9q22.2	rs7037744	Recessive	1.6 (0.5)
Colon	10q22.1	rs736594	Recessive	1.1 (0.4)
Colon	12q23.1	rs17290272	Recessive	1.4 (0.4)
Colon	14q31.3	rs981270	Recessive	1.6 (0.5)
Colon	16q12.2	rs1990637	Recessive	1.3 (0.4)
Colon	Xp22.31	rs1159561	Recessive	1.7 (0.8)
Rectal	2q23.3	rs1441973	Dominant	1.3 (0.7)
Rectal	6q24.3	rs6570867	Dominant	1.3 (0.7)
Rectal	9q22.33	rs1167768	Dominant	1.7 (0.9)
Rectal	10q26.2	rs1926143	Dominant	1.2 (0.7)
Rectal	18p11.2	rs872906	Recessive	2.6 (0.6)
Rectal	1q31.1	rs1160832	Recessive	1.4 (0.5)
Rectal	7q33	rs2059367	Recessive	1.1 (0.3)
Rectal	Xq26.3	rs2254857	Recessive	1.0 (0.4)

### Exome sequencing revealed genetic variants segregating in cancer families

In order to identify variants that possibly affect gene function in the linked regions, we did exome sequencing on twelve individuals representing the families contributing to the LOD scores. Three colon cancer families were included to investigate the candidate region on chromosome 6p21.1, whereas three rectal cancer families were included for the region on chromosome 18p11.2. Non-synonymous coding variants with a MAF < 0.20 in the regions of interest were assessed in relevant family members. We report variants segregating in all individuals for at least one of the three families (Tables 3 and 4). Twenty-two variants in 18 genes and 25 variants in 10 genes were identified segregating in colon cancer and rectal cancer families, respectively. Among the 22 variants observed in the colon cancer patients, 20 were missense variants, one was a frameshift insertion and one was an in-frame deletion. The 25 variants in the rectal cancer families were all missense variants.

**Table 3:** Sequence variants segregated in the colon cancer families

Genomic DNA change	cDNA change	rsID	Variant type	Gene	Amino acid change	MAF	Segregating families
NC_000006.11: g.38704943A>G	NM_001206927.1: c.863A>G	rs6935293	Missense	*DNAH8*	NP_001193856.1: p.(N288S)	0.06	110
NC_000006.11: g.38905957C>T	NM_001206927.1: c.11771C>T	rs61757218	Missense	*DNAH8*	NP_001193856.1: p.(T3924M)	<0.01	110
NC_000006.11: g.38980081A>G	NM_001206927.1: c.13462A>G	rs10484847	Missense	*DNAH8*	NP_001193856.1: p.(I4488V)	0.11	301
NC_000006.11: g.39034072G>A	NM_002062.4: c.502G>A	rs6923761	Missense	*GLP1R*	NP_002053.3: p.(G168S)	0.12	110,301
NC_000006.11: g.39507889C>T	NM_001289020.1: c.1535G>T	rs2273063	Missense	*KIF6*	NP_659464.3: p.(R512L)	0.08	301
NC_000006.11: g.40400288C>T	NM_020737.2: c.565G>A	rs61731040	Missense	*LRFN2*	NP_065788.1: p.(A189T)	0.07	110
NC_000006.11: g.41029342T>C	NM_006789.3: c.407T>C	rs2076472	Missense	*APOBEC2*	NP_006780.1: p.(I136T)	0.18	301, 350
NC_000006.11: g.41166017C>T	NM_024807.3: c.206G>A	rs77093113	Missense	*TREML2*	NP_079083.2: p.(R69Q)	<0.01	110, 301
NC_000006.11: g.41766439_41766443dup	NM_018561.4: c.1901_1905dup	rs201338884	frameshift ins	*USP49*	NP_061031.2: p.(D636fs)	0.01	301
NC_000006.11: g.41897919G>A	NM_004053.4: c.481G>A	–	Missense	*BYSL*	NP_004044.3: p.(E161K)	<0.01	301
NC_000006.11: g.41903798C>A	NM_001760.4: c.759G>T	rs33966734	Missense	*CCND3*	NP_001751.1: p.(E253D)	0.01	110
NC_000006.11: g.42932835C>T	ENST00000304611.8: c.2644G>A	rs141238034	Missense	*PEX6*	ENSP00000303511.8: p.(V882I)	0.02	301
NC_000006.11: g.42981051C>G	NM_014623.3: c.105G>C	rs35628750	Missense	*MEA1*	NP_055438.1: p.(E35D)	0.02	301
NC_000006.11: g.43013368C>T	NM_001168370.1: c.3071G>A	rs201130952	Missense	*CUL7*	NP_001161842.1: p.(R1024H)	<0.01	110
NC_000006.11: g.43014022G>A	NM_001168370.1: c.2864C>T	rs61732148	Missense	*CUL7*	NP_001161842.1: p.(A955V)	0.02	301
NC_000006.11: g.43034855C>T	NM_138343.3: c.913C>T	rs146188256	Missense	*KLC4*	NP_612352.1: p.(R305C)	0.01	301
NC_000006.11: g.43112267C>T	NM_001270398.1: c.2354C>T	rs34764696	Missense	*PTK7*	NP_001257327.1: p.(A785V)	0.03	110, 301
NC_000006.11: g.43153787G>A	NM_015089.3: c.845G>A	rs61743561	Missense	*CUL9*	NP_055904.1: p.(G282E)	0.01	110
NC_000006.11: g.43166414_43166416del	NM_015089.3: c.2871_2873delGGG	rs141674093	Inframe del	*CUL9*	NP_055904.1: p.(G958del)	0.04	110
NC_000006.11: g.43535018C>T	NM_020750.2: c.722G>A	rs34324334	Missense	*XPO5*	NP_065801.1: p.(S241N)	0.04	301
NC_000006.11: g.43596814C>G	NM_019096.4: c.86G>C	rs112851070	Missense	*GTPBP2*	NP_061969.3: p.(G29A)	0.02	110
NC_000006.11: g.44147821A>G	NM_007058.3: c.1561A>G	rs34710081	Missense	*CAPN11*	NP_008989.2: p.(I521V)	0.19	110, 301

Genomic coordinates were based on GRCh37 (hg19). MAF, minor allele frequency (based on the 1000Genomes project and the ExAC database when 1000Genomes data were not available)

**Table 4:** Sequence variants segregated in the rectal cancer families

Genomic DNA change	cDNA change	rsID	Variant type	Gene	Amino acid change	MAF	Segregating families
NC_000018.9:g.9944960A>G	NM_003574.5:c.457A>G	rs29132	Missense	*VAPA*	NP_003565.4:p.(M153V)	0.08	8
NC_000018.9:g.10471732G>A	NM_153000.4:c.448G>A	rs3748415	Missense	*APCDD1*	NP_694545.1:p.(V150I)	0.1	8, 918
NC_000018.9:g.10681711C>T	NM_022068.3:c.7387G>A	rs3748428	Missense	*PIEZO2*	NP_071351.2:p.(V2463I)	0.12	8
NC_000018.9:g.10699129G>T	NM_022068.3:c.6149C>A	rs113682091	Missense	*PIEZO2*	NP_071351.2:p.(A2050D)	0.1	8
NC_000018.9:g.10731428C>T	NM_022068.3:c.4832G>A	rs2865121	Missense	*PIEZO2*	NP_071351.2:p.(R1611Q)	0.16	918, 1213
NC_000018.9:g.10759840C>A	NM_022068.3:c.3443G>T	rs35033671	Missense	*PIEZO2*	NP_071351.2:p.(C1148F)	0.16	8
NC_000018.9:g.13008497G>C	NM_032142.3:c.333G>C	rs149216711	Missense	*CEP192*	NP_115518.3:p.(L111F)	0.02	1213
NC_000018.9:g.13055915A>C	NM_032142.3:c.3326A>C	rs11080623	Missense	*CEP192*	NP_115518.3:p.(Q1109P)	0.01	8
NC_000018.9:g.13056682G>A	NM_032142.3:c.4093G>A	rs2282542	Missense	*CEP192*	NP_115518.3:p.(V1365M)	0.19	918
NC_000018.9:g.13068109G>A	NM_032142.3:c.4631G>A	rs7228940	Missense	*CEP192*	NP_115518.3:p.(R1544H)	0.19	918
NC_000018.9:g.13095591G>A	NM_032142.3:c.6344G>A	rs56913743	Missense	*CEP192*	NP_115518.3:p.(R2115Q)	0.19	918
NC_000018.9:g.13100326G>A	NM_032142.3:c.6686G>A	rs74340616	Missense	*CEP192*	NP_115518.3:p.(R2229Q)	0.02	1213
NC_000018.9:g.13826678C>T	NM_005913.2:c.914C>T	rs143262370	Missense	*MC5R*	NP_005904.1:p.(T305I)	0.0024	1213
NC_000018.9:g.14537970C>T	NM_001137671.1:c.640G>A	rs148283099	Missense	*POTEC*	NP_001131143.1:p.(V214I)	0.05	8, 918
NC_000018.9:g.14542648C>A	NM_001137671.1:c.498G>T	rs12454500	Missense	*POTEC*	NP_001131143.1:p.(M166I)	0.16	918
NC_000018.9:g.14542931C>T	NM_001137671.1:c.215G>A	rs45554841	Missense	*POTEC*	NP_001131143.1:p.(C72Y)	0.17	1213
NC_000018.9:g.14542949T>C	NM_001137671.1:c.197A>G	rs9807555	Missense	*POTEC*	NP_001131143.1:p.(H66R)	0.18	1213
NC_000018.9:g.14543039T>C	NM_001137671.1:c.107A>G	rs45570841	Missense	*POTEC*	NP_001131143.1:p.(K36R)	0.006	8
NC_000018.9:g.14543063A>C	NM_001137671.1:c.83T>G	rs45626231	Missense	*POTEC*	NP_001131143.1:p.(F28C)	0.01	918
NC_000018.9:g.18534948G>C	NM_005406.2:c.3649C>G	rs201390233	Missense	*ROCK1*	NP_005397.1:p.(Q1217E)	0.07	8
NC_000018.9:g.19995731T>C	NM_172241.2:c.2044A>G	rs9946136	Missense	*CTAGE1*	NP_758441.2:p.(I682V)	0.19	1213
NC_000018.9:g.19996805T>C	NM_172241.2:c.970A>G	rs12961009	Missense	*CTAGE1*	NP_758441.2:p.(I324V)	0.02	1213
NC_000018.9:g.21124945C>G	NM_000271.4:c.1926G>C	rs1788799	Missense	*NPC1*	NP_000262.2:p.(M642I)	0.83^a^	918, 1213
NC_000018.9:g.21424991C>A	NM_198129.2:c.3622C>A	rs17202961	Missense	*LAMA3*	NP_937762.2:p.(P1208T)	0.05	1213
NC_000018.9:g.21511034C>A	NM_198129.2:c.8445C>A	rs1154232	Missense	*LAMA3*	NP_937762.2: p.(N2815K)	0.16	8

Genomic coordinates were based on GRCh37 (hg19). MAF, minor allele frequency (based on the 1000Genomes project and the ExAC database when 1000Genomes data were not available)

^a^It is the reference minor allele (NC_000018.9:g.21124945C) that segragates in the rectal cancer families

### Haplotype association analysis identified candidate targets

**Fig. 2:**
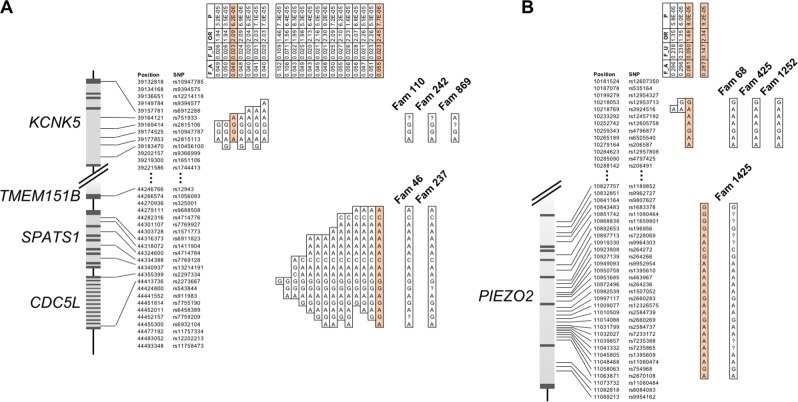
Candidate risk haplotypes revealed by sliding-window association analysis within the linked regions on chromosome 6 (**a**) and chromosome 18 (**b**). Association was evaluated for haplotypes of sizes ranging from 1 to 25 markers between 477 familial CRC cases and 4 780 controls. All haplotypes with OR > 1 and *p*-value < 1E−4 were listed with *p*-value, odds ratio (OR), estimated frequency in controls (F_U) and cases (F_A). One haplotype of highest interest (lowest *p*-value and highest OR) for each of the four loci was indicated in orange and searched among 60 CRC families. Familial haplotypes of the most informative families potentially carrying these haplotypes were listed (question marks indicate undetermined markers of the haplotypes). Genomic regions covered by these risk haplotypes were illustrated showing co-localized genes, where exons and introns were indicated with dark and light gray, respectively

To further pinpoint the genetic risk factors for colon and rectal cancers, we performed haplotype association analysis on the two regions of suggestive linkage (HLOD > 2). A total of 593 and 554 SNPs was successfully genotyped in the two regions on chromosomes 6 and 18, respectively. Association analysis between 477 familial CRC cases and 4780 controls using these genetic markers identified two candidate risk loci on chromosome 6 and two on chromosome 18. At least one candidate risk haplotype of each loci was associated with an elevated CRC risk (odds ratio 1.68–2.45) with a *p*-value lower than 1E−4. One of these four candidate risk haplotypes was relatively common (haplotype frequency of 15% in the control group), whereas the other three were infrequent (haplotype frequency 2–5% in the control group) (Fig. 2).

One risk haplotype identified on chromosome 6 stretched 14 kb in size, contained 4 markers and overlapped with gene *KCNK5* (Fig. 2a). Haplotyping of 60 CRC families revealed at least one colon cancer family (family 110), one rectal cancer family (family 242) and one CRC family without tumor site predominance (family 869) that potentially have this haplotype (Fig. 2a). It is notable that two of the three colon cancer families that contributed to the LOD score, families 110 and 301 (data not shown), were identified as potential carriers of this haplotype. The other risk haplotype on chromosome 6 was 176 kb in size and overlapped with genes *CDC5L*, *SPATS1* and part of *TMEM151B*. At least two colon cancer families (families 46 and 237) likely harbor this haplotype (Fig. 2a).

One risk haplotype on chromosome 18 was 61 kb in size but did not overlap with any known gene. Two rectal cancer families (families 425 and 1252) and one colon cancer family (family 68) in our study clearly harbored this haplotype (Fig. 2b). One of the most linked rectal cancer families, family 918, is also a potential carrier of this haplotype (data not shown). The other risk haplotype on chromosome 18 overlapped with part of the gene *PIEZO2*, and at least one rectal cancer family (family 1425) is likely to have this 220-kb haplotype based on genotyping of the parent–child pair (Fig. 2b).

## Discussion

CRC is a multifactorial disease. Previous studies have shown that tumor location differs among FAP compared to HNPCC patients and that different tumor sites would display diverse genetic alterations and allelic loss at 5q, 17p and 18 [19, 20]. Also, gene expressions and mutation rates vary among right and left colon and rectal tumors [21–23]. We hypothesized that, by subdividing the CRC families into colon and rectal cancer families, it would hopefully result in novel loci and predisposing genes for the two different cancer entities.

Our linkage analysis provided us with some interesting regions with suggestive linkage HLOD = 2.5 for the colon cancer families on locus 6p21.1-p12.1 and HLOD = 2.6 for the rectal cancer families on locus 18p11.2 (Table 2). These regions have not yet, to our knowledge, been reported by other linkage studies, possibly because no previous study subdivided the CRC families into colon cancer and rectal cancer families.

Exome sequencing was carried out on twelve individuals representing the families contributing to the LOD scores and identified within the linked regions 22 colon and 25 rectal cancer variants segregating in the cancer families, respectively (Tables 3, 4). Genes harboring these variants are involved in signal transduction (*GLP1R*, *CCND3*, *CUL7*, *PTK7*, *VAPA*, *APCDD1*, *MC5R*, *ROCK1*, *NPC1*), microtubule-based process (*DNAH8*, *KIF6*, *CUL9*, *CEP192*), RNA metabolism (*APOBEC2*, *USP49*, *BYSL*, *XPO5*), establishment of localization (*PEX6*, *GTPBP2* and *PIEZO2*) and cell differentiation (*LRFN2*, *MEA1*, *LAMA3*) among others. Some of these genes have been implicated in colorectal tumorigenesis, for instance, *CCND3* is a known oncogene in multiple cancer types including CRC [30]. *PTK7*, whose variants presented in two families, is reported to be expressed and actively involved in various malignancies including CRC, and its function in the Wnt signaling pathway has been demonstrated (reviewed in the ref. [31]). Moreover, overexpression of PTK7 has been implicated as a biomarker for adenoma and CRC, and is correlated with several clinicopathological features such as TNM stage, tumor differentiation, lymph node and distant metastasis [32, 33]. Similarly, GTPBP*2* is also known as a positive regulator of the Wnt signaling pathway [34], which is involved in tumorigenesis of a wide variety of cancers including CRC. The variant in the gene *APCDD1* is also shared among two families. APCDD1 is suggested to be regulated by the β-catenin/Tcf complex involved in colorectal tumorigenesis [35]. A previous methylation microarray-based scanning has revealed that hypermethylation of *GLP1R* is a biomarker for CRC and adenoma [36]. *CUL7* has been identified as an oncogene, since it could directly bind to p53 and prevent cells from Myc-induced apoptosis [37]. Overexpression of CUL7 could distinguish metastatic CRC samples from the non-metastatic ones [38]. XPO5 is a key protein responsible for miRNA transportation and is upregulated both at mRNA and protein levels in CRC. Its overexpression is associated with worse clinicopathologic features and poor survival in CRC [39]. The *POTEC* gene had one variant shared in two families and other variants in single families. *POTEC* is a member of the highly homologous POTE family which are expressed in multiple cancer types including colon cancer [40, 41]. Gene *ROCK1* is part of the Rho-kinase family and is overexpressed in CRC cell lines [42] and tissues [43]. Overexpression of ROCK1 has been shown to lead to increased CRC cell proliferation, transformation and invasion [42]. The gene *CTAGE1* is described as a cancer antigen for T-cell lymphoma and other malignancies [44], and is expressed in 12–19% CRCs [45]. Previous studies have reported somatic frameshift variants of *LAMA3* in CRC with high microsatellite instability [46] and deletions of the *LAMA3* gene in CRC with high chromosomal instability [47].

Haplotype analysis has been proven valuable in identifying susceptibility genes in familial breast cancer [48] and cancer syndromes [49], especially in populations with a relatively homogenous genetic background. In particular, a candidate CRC locus on chromosome 9q [8, 9, 13, 14] was recently suggested to be explained by two different risk haplotypes in familial and sporadic bowel cancer [50]. In order to search for additional support of the two loci in the current study and to further pinpoint candidate risk variants, we performed haplotype association studies between familial CRC cases and controls for the two regions. The four candidate haplotypes harbor coding regions of several genes including *CDC5L* (cell division cycle 5 like), a positive regulator of cell cycle G2/M progression and key promoter of colorectal cancer cells [51]. The relationship between colorectal cancer and other genes located within these candidate haplotypes haven’t been well studied. But the fact that some of the families in the linkage analysis were demonstrated to be potential carriers of these risk haplotypes supports that these haplotypes may by associated with an increased risk.

In conclusion, we propose two new linkage regions for colon cancer and rectal cancer. Haplotype analysis provides additional support and information regarding candidate variants that might affect function. We also report candidate variants within the linked regions that possibly predispose to CRC risk. Further studies on these genes of interest are needed to support or exclude them to be harboring disease causing variants.
